# Applying a bagging ensemble machine learning approach to predict functional outcome of schizophrenia with clinical symptoms and cognitive functions

**DOI:** 10.1038/s41598-021-86382-0

**Published:** 2021-03-25

**Authors:** Eugene Lin, Chieh-Hsin Lin, Hsien-Yuan Lane

**Affiliations:** 1 Department of Biostatistics, University of Washington, Seattle, WA 98195 USA; 2 Department of Electrical and Computer Engineering, University of Washington, Seattle, WA 98195 USA; 3 Graduate Institute of Biomedical Sciences, China Medical University, Taichung, Taiwan; 4 Department of Psychiatry, Kaohsiung Chang Gung Memorial Hospital, Chang Gung University College of Medicine, Kaohsiung, Taiwan; 5 School of Medicine, Chang Gung University, Taoyuan, Taiwan; 6 Department of Psychiatry, China Medical University Hospital, Taichung, Taiwan; 7 Brain Disease Research Center, China Medical University Hospital, Taichung, Taiwan; 8 Department of Psychology, College of Medical and Health Sciences, Asia University, Taichung, Taiwan

**Keywords:** Computational biology and bioinformatics, Cognitive neuroscience, Schizophrenia

## Abstract

It has been suggested that the relationship between cognitive function and functional outcome in schizophrenia is mediated by clinical symptoms, while functional outcome is assessed by the Quality of Life Scale (QLS) and the Global Assessment of Functioning (GAF) Scale. To determine the outcome assessed by QLS and GAF, we established a bagging ensemble framework with a feature selection algorithm resulting from the analysis of factors such as 3 clinical symptom scales and 11 cognitive function scores of 302 patients with schizophrenia in the Taiwanese population. We compared our bagging ensemble framework with other state-of-the-art algorithms such as multilayer feedforward neural networks, support vector machine, linear regression, and random forests. The analysis revealed that the bagging ensemble model with feature selection performed best among predictive models in predicting the QLS functional outcome by using 20-item Scale for the Assessment of Negative Symptoms (SANS20) and 17-item Hamilton Depression Rating Scale (HAMD17). Moreover, to predict the GAF outcome, the bagging ensemble model with feature selection performed best among predictive models by using SANS20 and the Positive and Negative Syndrome Scale-Positive (PANSS-Positive) subscale. The study indicates that there are synergistic effects between negative (SANS20) and depressive (HAMD17) symptoms as well as between negative and positive (PANSS-Positive) symptoms in influencing functional outcome of schizophrenia using the bagging ensemble framework with feature selection.

## Introduction

Functional outcome of schizophrenia, which is commonly assessed by the tools such as Quality of Life Scale (QLS)^1^ and the Global Assessment of Functioning (GAF) Scale^2^, has an impact on psychiatric diagnosis and treatment. In patients with schizophrenia, multiple functional domains, including work activities, social relationships, and independent living, of everyday life are usually impaired^3, 4^. Thereby, it is crucial to identify probable factors that influence functional outcome of schizophrenia^5^. Several potential predictors of its functional outcome include negative symptoms, verbal learning, visual learning, working memory, and social cognition, to name a few^3, 4, 6, 7^. GAF is recognized as an important objective measure to assess global psychological, social, and occupational functioning in patients with schizophrenia^2^. On the other hand, QLS is also useful in evaluating their functional outcome^8^. Thus, QLS and GAF have been used together for the assessment of longitudinal outcome of schizophrenia^9^. While some studies showed the limited predictive effect of clinical symptoms, such as positive symptoms^5, 10^, on functional outcome of schizophrenia, other studies indicated that clinical symptoms, particularly negative symptoms^11, 12^, were associated with its functional outcome. Moreover, it has been suggested that numerous cognitive functions such as neuro- and social-cognitions are also linked to its functional outcome^13, 14^. On another note, precision psychiatry is an emerging multidisciplinary arena of psychiatry and precision medicine^15, 16^, where state-of-the-art artificial intelligence and machine learning algorithms are incorporated with multiple data types such as genetic and clinical data to facilitate appropriate individual-tailored decisions during all stages of patient care^17–20^. For example, various applications in precision psychiatry encompass the prediction of patients with schizophrenia^21, 22^ and the prediction of antidepressant treatment outcome in patients with major depressive disorder^23, 24^ using machine learning models. We therefore proposed that machine learning models may be able to predict potential factors that affect functional outcomes of schizophrenia by using various clinical data (namely clinical symptoms and cognitive functions).

In a previous study, Lin et al.^5^ indicated that clinical symptoms mediated the relationship between cognitive impairment and functional outcome of schizophrenia by using the structural equation modeling method. Here, we utilized the same cohort of 302 patients with schizophrenia and carried out the first study on the QLS and GAF functional outcome prediction in schizophrenia patients with 3 clinical symptom scales and 11 cognitive function tests by using a bagging ensemble machine learning approach^25^. In addition, in order to forecast functional outcomes, we employed the M5 Prime feature selection algorithm^26^ to pinpoint a small subset of feasible factors from 3 clinical symptom scales and 11 cognitive function tests. We hypothesized that our bagging ensemble machine learning method would be able to predict the QLS- and GAF-related outcome in patients with schizophrenia by using a small subset of selected clinical symptom scales and/or cognitive function assessments. While no previous studies have evaluated predictive models for functional outcome of schizophrenia by using the bagging ensemble machine learning method with the M5 Prime feature selection algorithm, there have been studies that utilized the bagging and feature selection approaches generally for the prediction of functional outcome for individuals with psychosis^27, 28^. The bagging approach, which was created for simple bootstrapping in 1994, has been frequently utilized for experiments that employ a resampling scheme. We selected the bagging ensemble machine learning method since this method had been frequently applied to solve complex prediction and classification problems because of its advantages in reduction of variance and overfitting^25, 26^. This study directly compared the bagging ensemble machine learning model with widely-used machine learning algorithms, including multi-layer feedforward neural networks (MFNNs), support vector machine (SVM), linear regression, and random forests. We hypothesized that our bagging ensemble machine learning approach with the M5 Prime feature selection algorithm could lead to better performance.

## Results

### The clinical symptoms, cognitive manifestations, and functional outcome of the study cohort

The participants included 302 patients with schizophrenia in the Taiwanese population. Study measures relevant to their demographic characteristics, 3 clinical symptoms, 11 cognitive functions, QLS and GAF were detailed before^5^.

### Feature selection using clinical symptom scales

We performed a series of different feature combinations (Table 1; the Feature-A, Feature-B, and Feature-C sets) to predict the QLS and GAF scores using the 3 clinical symptom scales. Note that the Feature-A set includes the 3 clinical symptom scales, namely 17-item Hamilton Depression Rating Scale (HAMD17), 20-item Scale for the Assessment of Negative Symptoms (SANS20), and the Positive and Negative Syndrome Scale-Positive subscale (PANSS-Positive).

**Table 1:** The results of repeated tenfold cross-validation experiments for predicting the QLS and GAF functional outcome of schizophrenia with clinical symptom scales using machine learning predictors such as the bagging ensemble model with feature selection, the bagging ensemble model, MFNNs, SVM, linear regression, and random forests.

Algorithm	QLS			GAF		
	RMSE	Feature set	Number of features	RMSE	Feature set	Number of features
Bagging ensemble with feature selection	**6.4293 ± 1.1332**	Feature-B	2	**7.7806 ± 1.1595**	Feature-C	2
Bagging ensemble	6.4389 ± 1.1289	Feature-A	3	7.8133 ± 1.1758	Feature-A	3
SVM	6.4409 ± 1.1239	Feature-A	3	7.9147 ± 1.2053	Feature-A	3
MFNNs	6.4898 ± 1.0921	Feature-A	3	7.8432 ± 1.1721	Feature-A	3
Linear Regression	6.5616 ± 1.1660	Feature-A	3	7.9626 ± 1.2080	Feature-A	3
Random Forests	7.1563 ± 0.9873	Feature-A	3	8.4476 ± 1.2014	Feature-A	3

The best QLS or GAF scores are given in bold.

**Feature-A:** 3 features (related to 3 clinical symptom scales) including PANSS-Positive, SANS20, and HAMD17.

**Feature-B:** 2 features (related to 2 clinical symptom scales) including SANS20 and HAMD17.

**Feature-C:** 2 features (related to 2 clinical symptom scales) including PANSS-Positive, and SANS20.

GAF = Global Assessment of Functioning; HAMD17: 17-item Hamilton Depression Rating Scale; MFNNs = Multilayer Feedforward Neural Networks; PANSS-Positive: the Positive and Negative Syndrome Scale-Positive subscale; QLS = Quality of Life Scale; RMSE: Root Mean Square Error; SANS20: 20-item Scale for the Assessment of Negative Symptoms; SVM = Support Vector Machine.

Data are presented as mean ± standard deviation.

For predicting the QLS score, we used the M5 Prime feature selection algorithm (see Methods) to identify 2 features (including SANS20 and HAMD17) from the 3 clinical symptom scales, where these 2 chosen features comprised the Feature-B dataset.

For predicting the GAF score, we used the M5 Prime feature selection algorithm to find 2 features (including PANSS-Positive and SANS20) from the 3 clinical symptom scales, where these 2 chosen features comprised the Feature-C dataset.

### Prediction of QLS and GAF using clinical symptom scales

We combined clinical symptom scales (namely the Feature-A, Feature-B, and Feature-C datasets) to construct the predictive models for the QLS and GAF scores by employing the bagging ensemble framework, respectively. Table 1 summarizes the results of repeated tenfold cross-validation experiments for the predictive models using clinical symptom scales by the bagging ensemble model with feature selection, the bagging ensemble model, MFNNs, SVM, linear regression, and random forests. Moreover, we used the root mean square error (RMSE) values to measure the performance of the predictive models.

As indicated in Table 1, to predict the QLS, the bagging ensemble model with feature selection performed best in terms of the RMSE value of 6.4293 ± 1.1332 using the Feature-B dataset (namely SANS20 and HAMD17) among the predictive models. In other words, the combination of SANS20 and HAMD17 best predicted the QLS outcome among all combinations of clinical symptom scales.

In addition, to predict the GAF, the bagging ensemble model with feature selection performed best in terms of the RMSE value of 7.7806 ± 1.1595 using the Feature-C dataset (namely PANSS-Positive and SANS20) among the predictive models (Table 1). In other words, the combination of PANSS-Positive and SANS20 best predicted the GAF among all combinations of clinical symptom scales.

### Feature selection using cognitive function scores

We performed various feature combinations (Table 2; the Feature-D, Feature-E, and Feature-F datasets) to predict the QLS and GAF of schizophrenia using the cognitive function scores. Note that the Feature-D set included the 11 cognitive function scores.

**Table 2:** The results of repeated tenfold cross-validation experiments for predicting the QLS and GAF functional outcome of schizophrenia with cognitive function scores using machine learning predictors such as the bagging ensemble model with feature selection, the bagging ensemble model, MFNNs, SVM, linear regression, and random forests.

Algorithm	QLS			GAF		
	RMSE	Feature	Number of features	RMSE	Feature	Number of features
Bagging ensemble with feature selection	**7.7717 ± 1.0024**	Feature-E	5	**8.6050 ± 1.1101**	Feature-F	5
Bagging ensemble	7.8884 ± 1.0193	Feature-D	11	8.7804 ± 1.1481	Feature-D	11
SVM	7.9130 ± 1.0718	Feature-D	11	8.8969 ± 1.2218	Feature-D	11
MFNNs	7.9201 ± 0.9925	Feature-D	11	8.7932 ± 1.1239	Feature-D	11
Linear Regression	8.2865 ± 1.0381	Feature-D	11	9.0873 ± 1.2258	Feature-D	11
Random Forests	7.9133 ± 0.9795	Feature-D	11	8.9325 ± 1.1750	Feature-D	11

The best QLS or GAF scores are given in bold.

**Feature-D**: 11 features (related to 11 cognitive function scores) including category fluency, trail making A, WAIS-III digit symbol-coding, d-Prime of clear version, d-Prime of blurred version, verbal working memory, nonverbal working memory, verbal learning and memory, visual learning and memory, reasoning and problem solving, and social cognition.

**Feature-E**: 5 features (related to 5 cognitive function scores) including category fluency, WAIS-III digit symbol-coding, verbal working memory, nonverbal working memory, and social cognition.

**Feature-F**: 5 features (related to 5 cognitive function scores) including category fluency, WAIS-III digit symbol-coding, d-Prime of blurred version, verbal working memory, and reasoning and problem solving.

GAF = Global Assessment of Functioning; MFNNs = Multilayer Feedforward Neural Networks; QLS = Quality of Life Scale; RMSE: Root Mean Square Error; SVM = Support Vector Machine.

Data are presented as mean ± standard deviation.

For predicting the QLS, we used the M5 Prime feature selection algorithm (see Methods) to identify 5 features (including category fluency, WAIS-III digit symbol-coding, verbal working memory, nonverbal working memory, and social cognition) from the 11 cognitive function scores, where these 5 chosen features comprised the Feature-E dataset.

For predicting the GAF, we used the M5 Prime feature selection algorithm to find 5 features (including category fluency, WAIS-III digit symbol-coding, d-Prime of blurred version, verbal working memory, and reasoning and problem solving) from the 11 cognitive function scores, where these 5 selected features comprised the Feature-F dataset.

### Prediction of the QLS and GAF of schizophrenia using cognitive function scores

We used cognitive function scores (namely the Feature-D, Feature-E, and Feature-F datasets) to construct the predictive models for the QLS and GAF scores by employing the bagging ensemble framework, respectively. Table 2 summarizes the results of repeated tenfold cross-validation experiments for the predictive models using cognitive function scores by the bagging ensemble model with feature selection, the bagging ensemble model, MFNNs, SVM, linear regression, and random forests.

As shown in Table 2, to predict the QLS, the bagging ensemble model with feature selection performed best in terms of the RMSE value of 7.7717 ± 1.0024 using the Feature-E dataset (including category fluency, WAIS-III digit symbol-coding, verbal working memory, nonverbal working memory, and social cognition) among the predictive models. In other words, among all combinations of cognitive tests, the combination of category fluency, WAIS-III digit symbol-coding, verbal working memory, nonverbal working memory, and social cognition best predicted the QLS score.

In addition, to predict the GAF, the bagging ensemble model with feature selection performed best in terms of the RMSE value of 8.6050 ± 1.1101 using the Feature-F dataset (including category fluency, WAIS-III digit symbol-coding, d-Prime of blurred version, verbal working memory, and reasoning and problem solving) among the predictive models (Table 2). In other words, among all combinations of cognitive tests, the combination of category fluency, WAIS-III digit symbol-coding, d-Prime of blurred version, verbal working memory, and reasoning and problem solving best predicted the GAF score.

### Benchmarking

By comparing the results (Tables 1 and 2) for predicting the QLS of schizophrenia patients among machine learning predictive algorithms (including the bagging ensemble model with feature selection, the bagging ensemble model, MFNNs, SVM, linear regression, and random forests) using 4 feature datasets (including Feature-A, Feature-B, Feature-D, and Feature-E), the bagging ensemble model with feature selection (using Feature-B) performed best. The best RMSE value for predicting the QLS was 6.4293 ± 1.1332 (Table 1). In other words, the combination of SANS20 and HAMD17 best predicted the QLS performance among all clinical combinations and cognitive combinations.

By comparing the results (Tables 1 and 2) for predicting the GAF of schizophrenia among machine learning predictive algorithms (including the bagging ensemble model with feature selection, the bagging ensemble model, MFNNs, SVM, linear regression, and random forests) using 4 feature datasets (including Feature-A, Feature-C, Feature-D, and Feature-F), the bagging ensemble model with feature selection (using Feature-C) performed best. The best RMSE value for predicting the GAF was 7.7806 ± 1.1595 (Table 1). In other words, the combination of PANSS-Positive and SANS20 best predicted the GAF score among all clinical combinations and cognitive combinations.

Here, we found that the bagging ensemble model with feature selection using the selected features from clinical symptom scales performed best in predicting the QLS or GAF outcome when compared with other state-of-the-art algorithms, including MFNNs, SVM, linear regression, and random forests. Our analysis indicated that the bagging ensemble model with feature selection was well-suited for predictive models in the functional outcome of schizophrenia.

## Discussion

To our knowledge, this is the first study to date to identify synergistic effects between SANS20 and HAMD17 as well as between PANSS-Positive and SANS20 in influencing functional outcomes in schizophrenia among Taiwanese individuals using a bagging ensemble machine learning approach with the M5 Prime feature selection algorithm. Moreover, we performed the first study to predict potential factors affecting functional outcome of schizophrenia by utilizing various clinical data (that is, clinical symptoms and cognitive functions). The findings pinpointed that the bagging ensemble model with feature selection using 2 factors excelled other state-of-the-art predictive models in terms of RMSE for predicting the QLS outcome of schizophrenia, where these 2 factors encompassed SANS20 and HAMD17. Moreover, for predicting the GAF of schizophrenia patients, we found that the bagging ensemble model with feature selection using 2 factors outperformed other state-of-the-art predictive models in terms of RMSE, where these 2 factors encompassed PANSS-Positive and SANS20.

Interestingly, our analysis revealed that the combination of SANS20 (for measuring negative symptoms) and HAMD17 (for measuring depressive symptoms) was the best predictor for the QLS functional outcome of schizophrenia among all clinical symptom combinations and cognitive function combinations. In addition, the combination of SANS20 (for negative symptoms) and PANSS-Positive (for positive symptoms) was the best predictor for the GAF functional outcome of schizophrenia among all clinical symptom combinations and cognitive function combinations. In other words, there are synergistic effects between negative and depressive symptoms as well as between negative and positive symptoms in influencing functional outcome of schizophrenia. To the best of our knowledge, no previous studies have been conducted to identify a synergistic benefit beyond that of either clinical symptom scale standing alone. The interaction effects of clinical symptoms remain to be elucidated. It has been suggested that negative symptoms may act as a key predictor for functional outcome of schizophrenia^5, 29, 30^; moreover, positive symptoms may contribute to the GAF functional outcome^5, 31^. In addition, it has been suggested that depressive symptoms were related to the QLS performance^5, 30^. In consideration with the previous results^5, 29–31^, we speculated that SAN20 (for measuring negative symptoms) may likely incorporate with other factors such as HAMD17 (for depressive symptoms) or PANSS-Positive (for positive symptoms) to influence functional outcome of schizophrenia since all clinical symptoms are important predictors for functional outcome of schizophrenia.

By leveraging the clinical data, we established the predictive models of functional outcome of schizophrenia by using the bagging ensemble machine learning approach with the M5 Prime feature selection algorithm. Our analysis also suggests that the bagging ensemble model with feature selection may offer a feasible solution to construct predictive models for forecasting functional outcome of schizophrenia with purposeful accuracy. Therefore, the bagging ensemble approach with feature selection in this study is a proof-of-concept machine learning tool for predicting functional outcome of schizophrenia.

Furthermore, it is worthwhile to bring the discussion on the M5 Prime feature selection algorithm for dealing with potential factors affecting functional outcome of schizophrenia in our study. We observed that the bagging ensemble model with the chosen factors of the M5 Prime feature selection algorithm always surpassed the bagging ensemble model without using feature selection. For instance, the bagging ensemble model with the Feature-B dataset outperformed the bagging ensemble model with the Feature-A in predicting the QLS. Similarly, the bagging ensemble model with the Feature-C dataset outperformed the bagging ensemble model with the Feature-A dataset in predicting the GAF. That is, the bagging ensemble models with feature selection tended to have lower RMSE values. In terms of the predictive performance, the lower the RMSE value, the better the performance. We speculated that it may be due to the advantage of the M5 Prime feature selection algorithm to pinpoint probable factors influencing functional outcome of schizophrenia. In line with our analysis, previous studies indicated that machine learning algorithms with feature selection performed better than the ones without feature selection in forecasting disease status or treatment response for psychiatric disorders^24, 32, 33^.

Remarkably, an intriguing finding was that any of the machine learning models with clinical symptom scales always surpassed any of the machine learning models with cognitive function scores. For example, the predictive models with the Feature-A and Feature-B datasets always excelled the predictive models with the Feature-D and Feature-E datasets in predicting the QLS. Similarly, the predictive models with the Feature-A and Feature-C datasets always outperformed the predictive models with the Feature-D and Feature-F datasets in predicting the GAF. We hypothesized that it may be due to the advantage of clinical symptom scales over cognitive function scores on affecting functional outcomes in schizophrenia. In accordance with our analysis, it has been reported that clinical symptoms and cognitive functions accounted for 89% and 44% of the variance in the functional outcome of schizophrenia, respectively^5^.

This study had some limitations. The first weakness was that the cross-sectional methodology limited the predictive value. Second, both QLS and GAF scores were related to clinical symptoms, thereby causing an overlap between the predictors and the outcome.

In conclusion, we constructed a bagging ensemble machine learning framework with feature selection for estimating functional outcomes in schizophrenia in Taiwanese subjects by using clinical data. The analysis indicates that our bagging ensemble machine learning framework with feature selection detects synergistic effects between negative and depressive symptoms as well as between negative and positive symptoms in influencing functional outcomes in schizophrenia. In the long run, we would expect that the discoveries of the present study may be generalized for precision psychiatry studies to forecast functional outcome and disease status for psychiatric disorders. Moreover, the discoveries may be likely utilized to contribute to prognostic and diagnostic applications in the near future. All in all, it is indispensable to investigate in independent studies with replication samples and further explore the role of the bagging ensemble machine learning framework created in the present study.

## Materials and methods

This study was approved by the institutional review board of the China Medical University Hospital in Taiwan and was conducted in accordance with the Declaration of Helsinki.

### Study population

The study cohort consisted of 302 patients with schizophrenia, who were recruited from the China Medical University Hospital and affiliated Taichung Chin-Ho Hospital in Taiwan^5^. In this study, patients with schizophrenia were aged 18–65 years and were healthy in the physical conditions. After presenting a complete description of this study to the subjects, we obtained written informed consents from a parent and/or legal guardian in line with the institutional review board guidelines. Details of the diagnosis of schizophrenia were published previously^5^.

### Clinical symptom scales

In this study, we employed 3 clinical symptom scales to assess positive, negative and depressive symptoms^5^, including the PANSS-Positive subscale^34^, SANS20^35^, and HAMD17^36^.

### Cognitive function scores

We employed 11 cognitive function scores to assess cognitive functions^5^, including category fluency, trail making A, digit symbol-coding (Wechsler Adult Intelligence Scale, third edition (WAIS-III)), d-Prime of clear version, d-Prime of blurred version, verbal working memory, nonverbal working memory, verbal learning and memory, visual learning and memory, reasoning and problem solving, and social cognition. In brief, these 11 cognitive function scores were used to assess 7 cognitive domains such as speed of processing, sustained attention, working memory, verbal learning and memory, visual learning and memory, reasoning and problem solving, and social cognition^5^. The speed of processing domain was assessed using category fluency, trail making A, and WAIS-III digit symbol-coding. The sustained attention domain was assessed using d-Prime of clear version and d-Prime of blurred version. The working memory domain was assessed using verbal working memory and nonverbal working memory.

### Functional outcomes

We measured functional outcomes using the QLS^1^ and the GAF Scale of the DSM-IV^2^. QLS is a tool to provide the rating of functional outcomes in schizophrenia, including social activity, social initiatives, social withdrawal, sense of purpose, motivation, curiosity, anhedonia, aimless inactivity, capacity for empathy, emotional interaction^8^. GAF is a tool to provide a measure for assessing global psychological, social, and occupational functioning in schizophrenia^2^.

### Statistical analysis

The Student’s t test was conducted to measure the difference in the means of two continuous variables^37^. We performed the chi-square test for categorical data. The criterion for significance was set at *P* < 0.05 for all tests. Data are presented as the mean ± standard deviation.

With the assumption that a 95% confidence level and a proportion of 0.5, a simplified formula^38^ was used to calculate sample sizes as follows: *n* = *N* / (1 + *N* (*e*)^2^), where *n* is the sample size, *N* is the population size, and *e* is the level of precision. In this study, we assumed that *N* = 230,000 and *e* = 0.06.

### Bagging ensemble predictive models

We employed a key ensemble machine learning technique called bagging predictors^25^ and utilized the Waikato Environment for Knowledge Analysis (WEKA) software (which is available from https://www.cs.waikato.ac.nz/ml/weka/)^26^ to carry out the bagging ensemble predictive framework. In addition, other machine learning software tools can be employed, for example, Pattern Recognition for Neuroimaging Toolbox (PRoNTo; http://www.mlnl.cs.ucl.ac.uk/pronto/) and NeuroMiner (https://github.com/neurominer-git). All the experiments were conducted on a computer with Intel (R) Core (TM) i5-4210U, 4 GB RAM, and Windows 7^21^. It should be noted that we utilized the repeated tenfold cross-validation method to examine the generalization of bagging predictors^21, 32, 39^.

**Figure 1:**
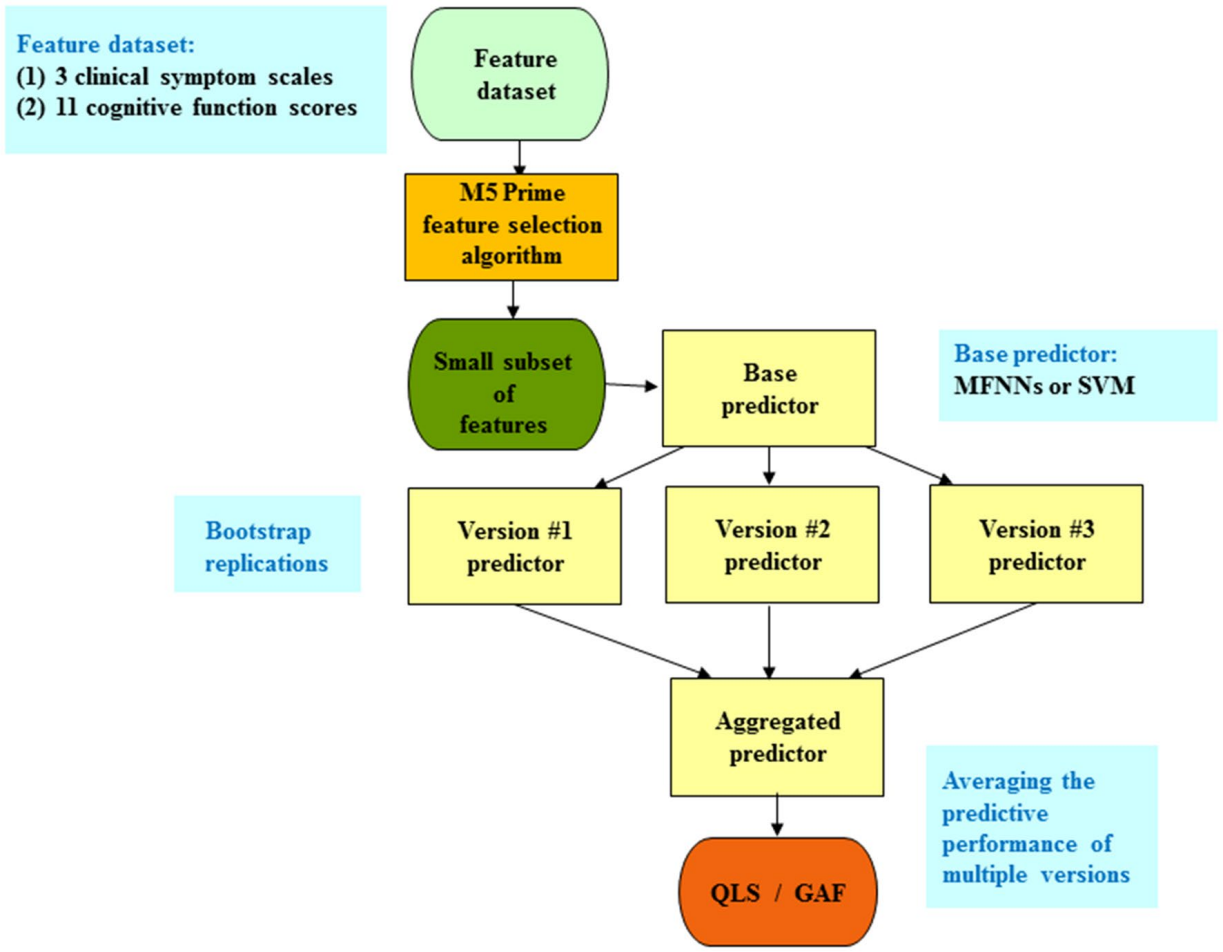
The schematic illustration of the bagging ensemble predictive algorithm with feature selection. First, the M5 Prime feature selection algorithm is performed to select a subset of features, which serves as the input to the bagging ensemble predictive algorithm. The idea of the bagging ensemble predictive algorithm is to generate the multiple versions of a base predictor by bootstrap replications. The final prediction is then produced by averaging the predictive performance of the multiple versions. The base predictor was chosen as multi-layer feedforward neural networks (MFNNs) or support vector machine (SVM) in this study. GAF = Global Assessment of Functioning; QLS = Quality of Life Scale.

Figure 1 shows the illustrative diagram of the bagging ensemble predictive framework with feature selection. The technique of the bagging ensemble predictive algorithm is used to combine the predictive performance of multiple versions of a base predictor to achieve an aggregated predictor with higher accuracy. The multiple versions of the base predictor are formed by the bootstrap method, where the bootstrap method is one of the most popular data resampling methods used in statistical analysis. The technique of the bagging ensemble predictive algorithm tends to reduce variance and avoid overfitting. The base predictor we employed was MFNNs or SVM. Here, we used the default parameters of WEKA, such as 100 for the batch size, 100 for the percentage of the bag size, and 10 for the number of iterations^21, 40^.

### Machine learning algorithms for benchmarking

For the benchmarking task in the present study, we utilized 4 state-of-the-art machine learning algorithms including MFNNs, SVM, linear regression, and random forests. We carried out the analyses for these 4 machine learning algorithms using the WEKA software^26^ and a computer with Intel (R) Core (TM) i5-4210U, 4 GB RAM, and Windows 7^21^. Other machine learning software tools such as PRoNTo (http://www.mlnl.cs.ucl.ac.uk/pronto/) and NeuroMiner (https://github.com/neurominer-git) could be also used. It should be noted that we utilized the repeated tenfold cross-validation method to examine the generalization of these 4 machine learning algorithms^21, 32, 39^.

An MFNN framework consists of one input layer, one or multiple hidden layers, and one output layer, where each layer contains neuron structures and connections among neuron structures contain no directed cycles^21, 41^. In general, the back-propagation algorithm^42^ is widely leveraged to train the MFNN framework, where the back-propagation algorithm updates the weights of neuron structures in the layers of the MFNN framework^21, 43^. In this study, we used the architecture containing 1 hidden layer. For example, we used the following WEKA’s parameters for training the MFNN model with 1 hidden layer: the momentum = 0.01, the learning rate = 0.01, and the batch size = 100^21, 40^.

The SVM algorithm^44^ is a popular technique for pattern recognition and classification^21^. The SVM algorithm, which is based on statistical learning theory, finds a linear relationship between input variables and the dependent variable (that is, the predicted output)^44, 45^. The best model for the predicted output is obtained by minimizing both the coefficients of the cost function and the predictive errors, where the cost function consists of the regression coefficients and an error term^44, 45^. In this study, we used the polynomial kernel with the exponent value of 1.0^24^.

The random forests model combines a collection of decision trees, where a decision tree is defined as an inverted tree with three types of nodes such as a root node, internal nodes, and leaf nodes^21, 46^. The random forests model is conceptualized to obtain a better prediction by aggregating the predictive results from a collection of decision trees^21, 46^. Here, we used the default parameters of WEKA for the random forests model; for example, 100 for the batch size and 100 for the number of iterations^21^.

The linear regression model, the standard method for prediction problems in clinical applications, was used as a basis for comparison^21, 26^. Linear regression is suitable for assessing the relationship between a scalar response (that is, a dependent variable) and explanatory variables (that is, independent variables) by fitting a linear equation to the data ^21, 26^.

### M5 Prime feature selection algorithm

In the present study, we utilized an Akaike information criterion (AIC)-based approach called the M5 Prime algorithm^26^ for the feature selection task. The M5 Prime algorithm constructs a decision tree with multivariate linear models at the terminal nodes and iteratively removes the feature with the smallest standardized coefficient until no further improvement in the estimated error defined by the AIC^47, 48^. Moreover, we utilized the tenfold cross-validation method to examine the generalization of the feature selection task^21, 32, 39^.

To predict the QLS and GAF, we used the M5 Prime algorithm to select features from 2 different feature datasets (Fig. 1). The first feature dataset includes 3 clinical symptom scales. The second feature dataset includes 11 cognitive function scores.

### Evaluation of the predictive performance

In this study, we utilized one of the most popular criteria, the RMSE, to assess the performance of predictive models^32, 45, 49^. The RMSE calculates the difference between the measured values and the estimated values by a predictive model. The better the prediction model, the lower the RMSE^32, 49^. Moreover, we utilized the repeated tenfold cross-validation method to examine the generalization of predictive models^21, 32, 39^. First, the whole dataset was randomly split into ten separate segments. Second, the predictive model was trained using nine-tenths of the data and was tested using the remaining tenth of data to evaluate the predictive performance. Next, the previous step was repeated nine more times by leaving out distinct nine-tenths of the data as training data and a distinct tenth of data as testing data. Finally, the average estimation was reported over all runs by processing the aforementioned tenfold cross-validation for 10 times with distinct batches of data. We estimated the performance of all predictive models using the repeated tenfold cross-validation method.
